# Diverse Patescibacteria assemblages and prevalence of ultra-small free-living Parcubacteria along a subterranean estuary

**DOI:** 10.1128/msystems.01125-25

**Published:** 2025-10-20

**Authors:** Clara Ruiz-González, Catalina Mena, Francisco M. Cornejo-Castillo, Daniel Romano-Gude, Néstor Arandia-Gorostidi, Josep M. Gasol

**Affiliations:** 1 Institut de Ciències del Mar (ICM-CSIC) 58341, Barcelona, Spain; 2 Instituto Español de Oceanografía (IEO, CSIC), Palma de Mallorca, Spain; University of Hawaii at Manoa, Kaneohe, Hawaii, USA

**Keywords:** subterranean estuary, coastal aquifer, Patescibacteria, Parcubacteria, Candidate Phyla Radiation, ultra-small prokaryotes

## Abstract

Patescibacteria are a group of novel, mostly uncultivated bacteria characterized by ultra-small cell sizes and streamlined genomes. They are ubiquitous in diverse ecosystems, often prevailing in subsurface environments, yet basic aspects such as variability in cell size, abundance, and niche preferences of different taxa within Patescibacteria remain unknown, particularly along salinity gradients. Combining flow cytometry, catalyzed reporter deposition-fluorescence *in situ* hybridization (CARD-FISH), and 16S rRNA gene sequencing, we characterized Patescibacteria assemblages along a Mediterranean subterranean estuary resulting from the mixing of fresh groundwater with seawater. Patescibacteria occupied the entire subterranean salinity gradient through the replacement of taxa prevailing in fresh (Magasanikbacteria, Jorgensenbacteria UBA9983), brackish (Portnoybacteria, Yanosfkybacteria, and Peribacteria), and saline groundwater (Nomurabacteria, unidentified Gracilibacteria). Most of the detected ASVs showed less than 95% similarity to their closest match, pointing to high novelty within coastal groundwater Patescibacteria. Flow cytometry unveiled a clear population of ultra-small prokaryotes that increased in abundance from fresh to saline groundwater, and which coincided with the presence of free-living minute coccoid cells identified as Parcubacteria by CARD-FISH. Some symbiotic-like associations with prokaryotes and eukaryotes were also observed, at least within Parcubacteria. These results provide one of the rare visual observations of Patescibacteria, and the substantial diversity of yet-unidentified taxa suggests an overlooked importance of this group in coastal groundwater.

**IMPORTANCE:**

Patescibacteria are an enigmatic group of bacteria of ultra-small sizes and reduced genomes, commonly found in subsurface environments but largely unexplored in terms of their ecological roles. Despite being present in both freshwater and marine systems, no study has explored how they distribute along salinity gradients. This study provides new insights into their distribution, diversity, and niche partitioning along a Mediterranean subterranean estuary characterized by a strong salinity gradient. We show that Patescibacteria taxa seem to adapt to varying groundwater salinity conditions, displaying a remarkable capacity to occupy fresh, brackish, and saline niches through changes in composition. The identification of ultra-small coccoid cells and symbiotic-like associations highlights a diversity of lifestyles within these groups and provides one of the scarce visual proofs of Patescibacteria. With most detected taxa being highly novel, these findings point to an overlooked importance of Patescibacteria in coastal aquifers, biogeochemically active sites ubiquitous along most coastlines.

## INTRODUCTION

The recent discovery of Patescibacteria, also known as Candidate Phyla Radiation or CPR, has largely expanded the tree of life by adding an enormous diversity of previously unknown groups (1–4). Many Patescibacteria are characterized by extremely small cell sizes (0.009 ± 0.002 µm^3^ [5, 6]) and very small genomes (~1 Mb), generally lacking common biosynthetic capabilities (i.e., production of amino acids, nucleotides, lipids, absence of respiratory genes [1, 4, 7–9]) and often harboring protein-coding gene families involved in pili and cell-cell interactions (6, 10–12). These features led to suggest that Patescibacteria have symbiotic or syntrophic lifestyles, depending on other microorganisms for certain metabolites while providing others to their hosts (2, 6, 13–19), and parasitic lifestyles have also been recently reported (20–23). However, some Patescibacterial groups are often enriched in the fraction of cells passing through the 0.2 µm filter (6, 11, 24, 25), indicating that some are either physically detached from their hosts or display a free-living lifestyle (11, 12, 26). Although extensive research using metagenomics or single-cell genomics has greatly advanced our knowledge on the phylogenetic diversity and metabolic potential of Patescibacteria (8, 15, 27–30), the general scarcity of visual information has prevented gaining further insight on basic features such as cell abundance, size variability (as not all members are equally small, e.g., 31), metabolic status, or their diversity of lifestyles in natural ecosystems.

Patescibacteria can be found in a variety of natural surface and subsurface habitats, including marine, freshwater, soil, permafrost, plant rhizosphere ecosystems, or animal-associated habitats (27, 29–37), and even in the air (32, 38), but they are usually most abundant in oligotrophic and oxygen-poor groundwater environments (1, 6, 8, 9, 11, 24). Accumulating evidence of biogeography within Patescibacteria shows that the relative abundance of its members varies over time or along groundwater flow paths (i) in response to variations in physicochemical parameters (6, 11, 12, 24, 39, 40), (ii) between different size fractions in groundwaters, surface freshwater, or seawater (6, 11, 12, 18, 24, 32, 41), (iii) due to the presence of other microorganisms (6, 11, 39, 42), and (iv) with differential connectivity of aquifers to the surface, as these groups seem to be preferentially mobilized from soils due to their small sizes (24, 39). To our knowledge, however, no study has explored the niche partitioning of Patescibacterial members along salinity gradients despite the relevance of salinity for prokaryotic community assembly (43). The belowground mixing of groundwater and seawater often results in pronounced salinity variations (i.e., the so-called subterranean estuaries [44]), and hence groundwater Patescibacteria may encounter steep salinity gradients during transit through coastal sediments. Therefore, coastal aquifers, which have been much less studied than inland groundwaters in terms of their microbiota (45), are unique systems for exploring salinity-driven niche partitioning in Patescibacteria.

Although Patescibacteria have rarely been reported in coastal aquifers (45), a few recent studies have detected their presence in shallow coastal groundwater of different salinities (46, 47). Similarly, in a coastal aquifer connected to the Mediterranean Sea, where groundwater was sampled at greater depths (6–22 m) than in most coastal surveys, we recently found that Patescibacteria represented the second most abundant phylum across the entire groundwater salinity gradient (48). Hence, here we aimed at exploring whether different Patescibacteria have different tolerances to salinity and/or other conditions within this system, where groundwater salinity ranges from 0.5 to 35 (48). Patescibacteria were identified by Illumina sequencing of the 16S rRNA gene, and flow cytometry, and epifluorescence and scanning electron microscopy (SEM) were used to visualize potentially ultra-small cells across the studied transect. Our results unveil a high diversity, novelty, and significant abundance of Patescibacteria along the entire subterranean salinity gradient and provide one of the scarce visual proofs of these enigmatic ultra-small bacteria.

## MATERIALS AND METHODS

### Study site and sample collection

**Fig 1:**
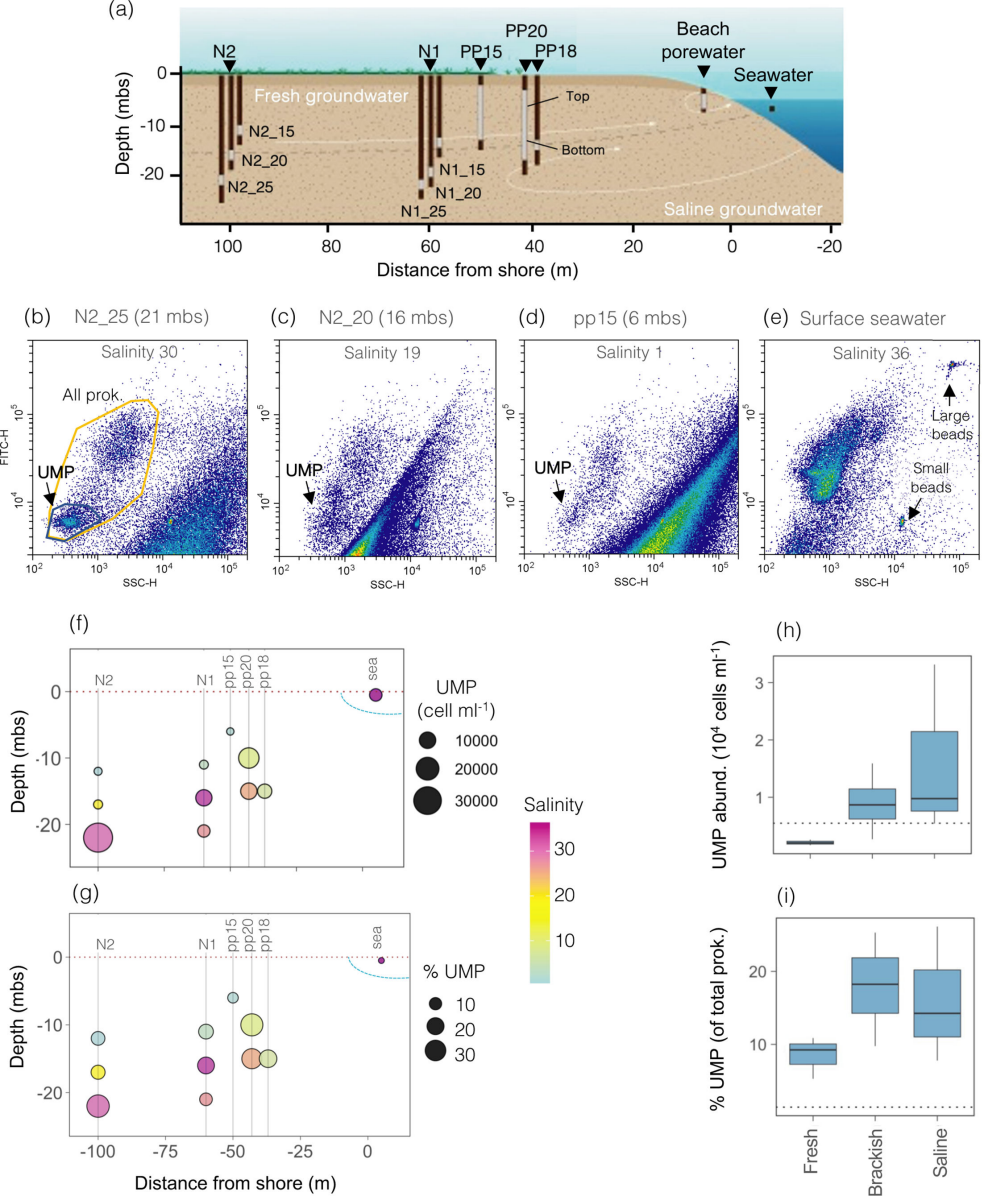
(**a**) Scheme showing the location of the collected samples at the experimental site in Argentona (modified from Ruiz-González et al. [48]). Shown are the piezometers (black bars) and the screened intervals (gray) of each piezometer (gray areas). Piezometers (*n* = 10) are distinguished from the beach porewater and the seawater samples. N1 and N2 refer to two nests consisting of three piezometers screened at different depths. (**b–e**) Flow cytometric plots of groundwater samples of different salinities (saline [**b**], brackish [**c**], fresh [**d**], and the seawater sample [**e**]) stained with SybrGreenI, highlighting all prokaryotes (yellow polygon) and the presence of the sub-population that was considered as ultra-small prokaryotes (UMP, black polygon). Note its close proximity to small fluorescent beads of 0.5 µm, whereas the large beads had 0.74 µm. FITC indicates green fluorescence intensity, SSC indicates side scatter. (**f and g**) Variations in the number (**f**) and percentage (**g**) of UMP across the study site quantified by flow cytometry, indicated by the size of the dot. The position of the sampled piezometers is indicated with vertical gray lines, as well as that of the seawater sample, and the color gradient indicates the salinity of each sample. (**h and i**) Variations in the abundance (**h**) or proportion (**i**) of UMP between the different types of groundwater considering only the aquifer samples (*n* = 10). The dotted line represents the UMP abundance or percentage in the seawater sample, respectively, for comparison. Mbs, meters below surface.

Details on the study site and sampling design are described in reference 48, and information on the hydrogeology and geology of the site can be found in references 49, 50. Briefly, the sampling was performed in July 2018 (representing mid-summer conditions) along a subterranean estuary connected to the Mediterranean Sea (the Argentona site, Barcelona, Spain) (Fig. 1a). The area is characterized by a Mediterranean temperate climate marked by warm, dry summers and wet, mild winters. Annual precipitation in the area ranges from 350 to 930 mm yr^−1^ (period 2015–2020) and is mainly governed by extreme precipitation episodes that take place during spring and autumn (51). In the aquifer, fresh groundwater flows seaward in the upper part, and the deeper area (<15 mbs) is affected by a seawater intrusion extending >150 m inland that is generally most evident in summer (49, 50). Groundwater was collected through a series of installed piezometers that allow sampling the entire salinity gradient at both the vertical and the horizontal scales. Ten groundwater samples were collected at depths ranging from 6 to 22 mbs (meters below the surface) and distances from the shore ranging from 37 to 100 m, covering the entire groundwater salinity gradient (0.5–33.6). Also, one beach porewater sample (salinity 35.2) and one surface seawater sample (36.4) were collected next to the shore line (Fig. 1a). For all sampling sites, temperature, dissolved oxygen, pH, and salinity were measured *in situ* with a YSI probe, and inorganic nutrient concentrations (nitrate, nitrite, ammonium, phosphate, and silicate) were determined with a SEAL AutoAnalyzer 3 (AAC HR, SEAL Analytical), coupled to a Jasco FP2020 for the determination of ammonia, as detailed in reference 48.

### Characterization of Patescibacteria communities

The taxonomic composition of Patescibacteria communities was determined from the DNA sequence data available at the European Nucleotide Database (ENA) under accession number PRJEB52186. Details on the DNA extraction and bioinformatic processing are provided in reference 48. Briefly, between 2.3 and 10 L of water were filtered onto 0.2 µm polycarbonate membrane filters (47 mm, Merck Millipore) using a peristaltic pump. DNA was extracted from the filters using the standard phenol-chloroform protocol with slight modifications (52). The V3–V4 region of the 16S rRNA gene was amplified with the general bacterial primers 341F and 805R (53), shown to capture a higher diversity of Patescibacteria than other commonly used primers (33, 45), and which have been applied in groundwater, freshwater, and marine surveys (24, 32, 33, 41). Amplicons were sequenced in an Illumina MiSeq platform following a paired-end approach at the RTLGenomics facility. Amplicon sequence variants (ASVs) were generated using DADA2 v.1.16 (54) and for this study, taxonomic assignment was performed using SILVA v.138.2 through the RDP naïve Bayesian classifier method (55). 1015 ASVs assigned to Patescibacteria were kept and used for further analyses.

### Novelty and phylogenetic reconstruction

In order to identify novel Patescibacteria, we analyzed the % of nucleotide identity shared between the 1,015 Patescibacteria ASVs and their closest match in the SILVA v138.2 database using VSEARCH v2.17 (56) in the “–usearch_global” mode with default parameters. For the phylogenetic reconstruction, a fasta nucleotide file containing a subset of the most abundant Patescibacteria ASVs (i.e., those comprising >0.1% in at least one sample, *n* = 459 ASVs) and one Archaea ASV (used as an outgroup in the phylogenetic tree) was aligned using Muscle (v3.8.425 [57]) with default parameters. The resulting alignment was used as input to perform a phylogenetic reconstruction based on maximum likelihood with RaxML(raxml-ng/0.9.0) (58), run with the following parameters: raxml-ng -all –model GTR + IO –bs-trees autoMRE. The phylogeny was visualized and rooted using anvi’o v8 (59).

### Abundances of ultra-small prokaryotes

Total free-living prokaryotic abundances were determined by flow cytometry as described in reference 48, and the abundance of ultra-small prokaryotes (hereafter UMP) was estimated by selecting a population on the cytograms based on the side scatter and fluorescence of the SybrGreenI (Fig. 1). Since the UMP population was most clearly defined in the deeper saline groundwater sites (Fig. 1b), we used these samples to delineate and quantify the UMP population across all samples. UMP from the beach porewater sample was not considered due to the presence of many small particles overlapping with the UMP flow cytogram area (details not shown).

### Visualization of Parcubacteria via catalyzed reporter deposition-fluorescence *in situ* hybridization (CARD-FISH)

The presence, abundance, and potential lifestyle (free-living or attached/symbiotic) of Parcubacteria (previously known as OD1), which was the most abundant Patescibacteria group, was explored via CARD-FISH applying the probe OD1-289 (14) to samples collected onto 0.2 µm filters, as detailed in reference 48. Counterstaining of CARD-FISH filters was done with 4,6-diamidino-2-phenylindole (DAPI), and the abundance of OD1 was estimated manually with an Olympus BX61 epifluorescence microscope counting a minimum of 10 fields (550–1,200 DAPI-stained cells). The specificity of the OD1 probe for Parcubacteria was tested *in silico* using the online tool TestProbe (60) by comparing the sequences from the SILVA taxonomic database that would perfectly match the OD1-289 probe sequence. Except for a single matched sequence of Gracilibacteria (Fig. S1), which was not detected among our ASVs, no unspecific binding with other groups was found, supporting that all OD1-hybridized cells belong to Parcubacteria although not all Parcubacterial groups were equally covered by the probe.

The size of the OD1-labeled cells of the three samples showing the highest OD1 abundances (N1_20, N1_25, and N2_25) was estimated by automated image acquisition using the epifluorescence microscope Axio Imager.Z2m connected to a Zeiss camera (AxioCam MRm, Carl Zeiss MicroImaging) at 630× magnification followed by automated image analysis using ACMEtool3 (M.Zeder, GmbH 2014). Image acquisition was performed with the AxioVision 4.8 software using the DAPI (DNA staining; UV excitation, 385 nm) and Alexa-488 (OD1-labeled cells; blue light excitation, 470 nm) channels. We used the area (in pixels) of the DAPI signal of OD1-labeled cells to avoid overestimation of cellular sizes due to differences in Alexa-488 fluorescence intensity. The size of 200–500 measured OD1 cell areas per sample was compared with those of SAR11-hybridized cells (a known marine ultramicrobacteria) from three samples of a nearby oligotrophic marine station (Blanes Bay Microbial Observatory, bbmo.icm.csic.es), hybridized with the probe SAR11-441R (61), using the same procedure and settings, and counting 700-1100 cells/sample.

### Scanning electron microscopy (SEM)

Samples for SEM were collected from N2_25 (highest abundance of UMPs). 1 L of water was sequentially filtered through 0.2 and 0.1 µm pore-size polycarbonate filters (47 mm, GTTP and VCTP, respectively, Merck Millipore). The 0.1 µm pore-size filter was fixed with glutaraldehyde 25% grade I for 1 h (2.5% final concentration) and subsequently dehydrated with increasing concentrations of ethanol in 15 min (20%, 30%, 50%, 70%, 80%, and 90%) and 30 min (95% and 100%) steps. A section of the filter was critical-point dried in a Bal-Tec CPD030 apparatus (Leica Microsystems, Vienna, Austria), mounted on a stub, sputter-coated with gold in a Quorum Q150RS (Quorum Technologies, Ltd, Laughton, UK) and inspected using a variable-pressure SEM (Hitachi S-3500N, Hitachi High Technologies Corporation, Japan) at the Servei de Microscòpia Electrònica (ICM-CSIC, Barcelona, Spain).

### Statistical analyses

The taxonomic richness of Patescibacteria was calculated using the total ASV table rarefied at 10,635 reads/sample (see [48]). The niche preference of different Patescibacteria ASVs was estimated by assigning to each ASV the habitat type (fresh, brackish or saline groundwater, beach porewater or surface seawater) where it showed its maximum relative abundance across the entire dataset. Heterogeneity in physicochemical variables (Euclidean distances) was compared to community dissimilarity (Bray-Curtis) using Mantel tests (mantel function, R vegan package). The effect of physicochemical variables on community structure was further assessed through redundancy analysis (RDA) with forward selection (vegan R package). Prior to RDA, community data were Hellinger-transformed and environmental variables were normalized by *z*-score. Forward selection included permutation-based ANOVA tests (999 permutations) at each step to identify significant variables (*P* < 0.05). Collinearity among significant variables was evaluated using variance inflation factors (VIFs), and only variables with VIF < 10 were retained and used to build the final RDA model. The relationship between the physico-chemical variables, and the relative abundance of taxonomic groups was estimated by means of Spearman correlations (R ggpubr) and the results of the correlation analysis were visualized in a heatmap (R ComplexHeatmap). All analyses were run using R software version 3.5.3 (62).

## RESULTS

### Abundances, size, and morphology of ultra-small prokaryotes

Flow cytometry unveiled a population of ultra-small cells in most aquifer samples that were not detected in the surface seawater sample (Fig. 1b through e). This population was most clearly defined in the two deepest saline samples N2_25 and N1_25 (Fig. 1b), so we chose those samples to delineate the population that we considered UMP and quantified it across the studied transect. Within the aquifer, UMP abundance increased with increasing salinity and ranged from 1.6 × 10^3^ cells mL^−1^ in the fresh groundwater site PP15 to a maximum abundance of 3.3 × 10^4^ cells mL^−1^ in the deep saline N2_25 site (Fig. 1f and h). UMP abundance covaried positively with that of total prokaryotes (*r* = 0.92, *P* < 0.001, *n* = 10), and represented between 5% and 26% of total prokaryotic counts throughout the aquifer (Fig. 1g and h). There was no obvious UMP population in the surface seawater sample (Fig. 1e).

### Taxonomic composition of Patescibacteria

**Fig 2:**
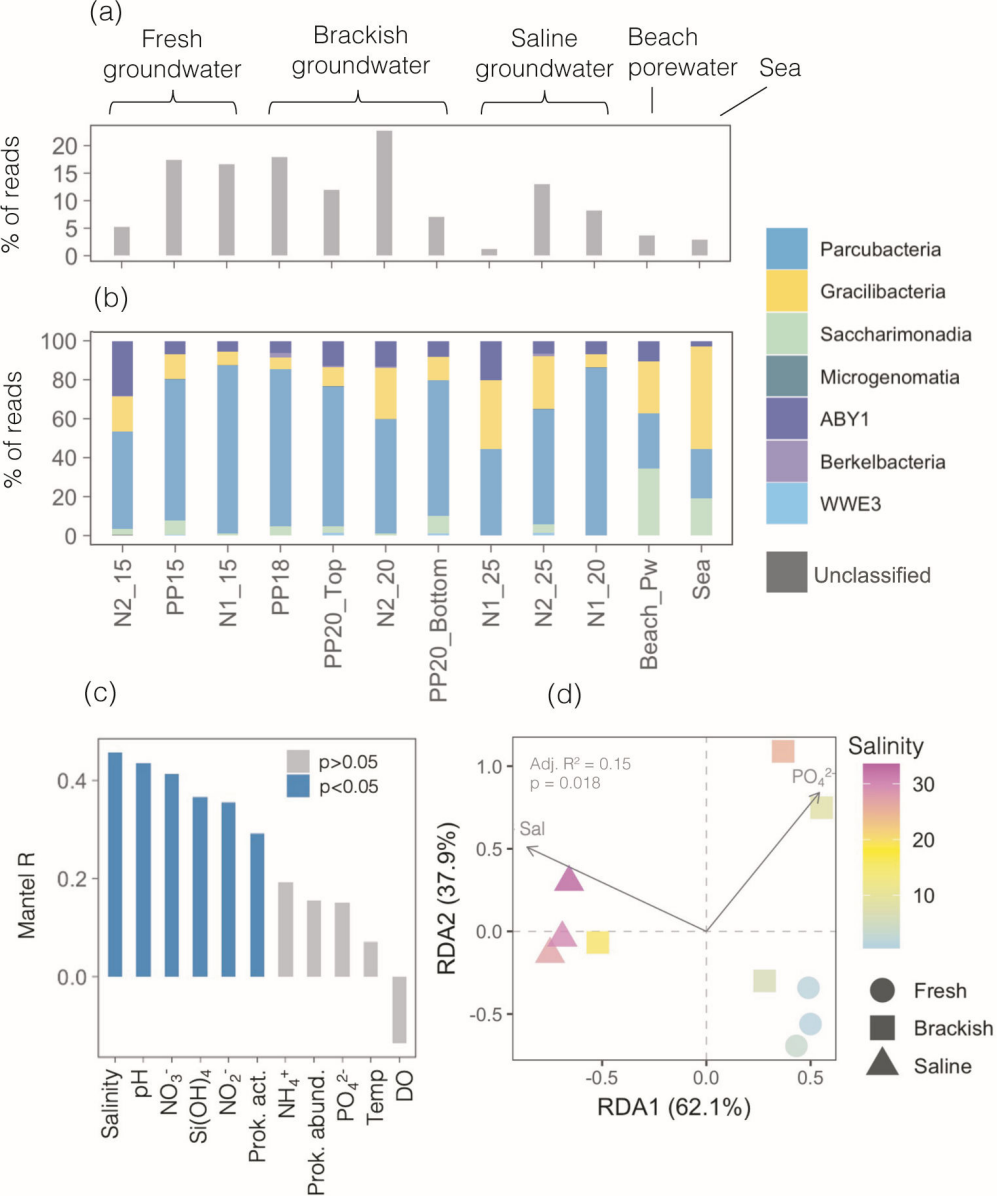
(**a**) Proportion of reads belonging to Patescibacteria across the studied samples, which are ordered by increasing salinity. (**b**) Taxonomic composition at the class level of the Patescibacteria assemblages. (**c**) R coefficients of the Mantel correlations between differences in environmental variables and Patescibacterial Bray-Curtis dissimilarity matrices, pooling all groundwater samples together (*n* = 10). (**d**) Redundancy analysis (RDA) of the Patescibacteria assemblages including only the two significant environmental variables selected by forward selection: salinity and phosphate. The adjusted *R*^2^ (Radj) and *P* value (ANOVA-like permutation test) represent the most parsimonious model. The color gradient indicates the salinity of each sample. Prok. act., prokaryotic heterotrophic activity estimated as radiolabelled leucine uptake; Prok. abund, total prokaryotic abundance; Temp, groundwater temperature; DO, dissolved oxygen concentration.

Patescibacteria represented the second most abundant phylum in the subterranean estuary after Proteobacteria (48). The relative contribution of the 1,015 ASVs identified as Patescibacteria ranged from 1.2% to 22% of sequences in aquifer communities, and contrary to the UMP abundances, was generally higher in fresh and brackish than in the saline groundwater sites (Fig. 2a). Less than 4% of the sequences in the beach porewater sample and the surface seawater were associated with Patescibacteria (Fig. 2a).

In terms of classes, Patescibacteria communities were dominated by the class Parcubacteria (74% of Patescibacterial reads, 537 ASVs), followed by Gracilibacteria (14%, 181 ASVs) ABY1 (9%, 147 ASVs), Saccharimonadia (3%), Berkelbacteria (0.5%), WWE3 (0.3%), Microgenomatia (0.15%), and other unidentified Patescibacteria (0.02%). However, a large fraction (76%) of the detected ASVs showed less than 95% similarity to their closest match on the SILVA database (Fig. S2), pointing to high novelty within the studied Patescibacterial assemblages. Based on the 16S rRNA partial sequence similarity thresholds proposed by Yarza et al. (63), >70% of the detected ASVs may represent potentially novel genera, whereas 27%, 9%, and 4% of the ASVs may be currently unknown families, orders, or classes, respectively (Fig. S2). Only seven ASVs were 100% identical to sequences present in the SILVA database.

Parcubacteria comprised between 45% and 86% of Patescibacteria sequences in groundwater (Fig. 2b) and showed no obvious variations along the salinity gradient. Aquifer Gracilibacteria (6–35%) showed higher abundances in some of the most saline sites and was the dominant class in the surface seawater sample (53%). Saccharimonadia ranged from 0% in N1_25 to 9% of the sequences within the aquifer, comprising up to 34% and 19% in the beach porewater and surface seawater, respectively. Finally, ABY1 accounted for 5–28% of the aquifer Patescibacteria, and only 3% of the seawater community (Fig. 2b).

The number of detected ASVs per sample ranged between 21 and 244 ASVs and decreased with increasing groundwater salinity (Fig. S3). Indeed, salinity was the variable most strongly correlating with taxonomic dissimilarity among Patescibacterial assemblages (Mantel *R* = 0.46, *P* < 0.05), followed by pH, nitrate, silicate, nitrate, and the activity of prokaryotic communities measured as ^3^H-leucine uptake (Fig. 2c). When included in the RDA, salinity was identified as the most significant environmental variable explaining community variance (*F* = 2.044, *P* = 0.004), followed by phosphate (*F* = 1.619, *P* = 0.013). Saline and fresh groundwater sites were clearly separated in the RDA, while brackish sites appeared to be more heterogeneous (Fig. 2d).

### Microscopic visualization of ultra-small prokaryotes

**Fig 3:**
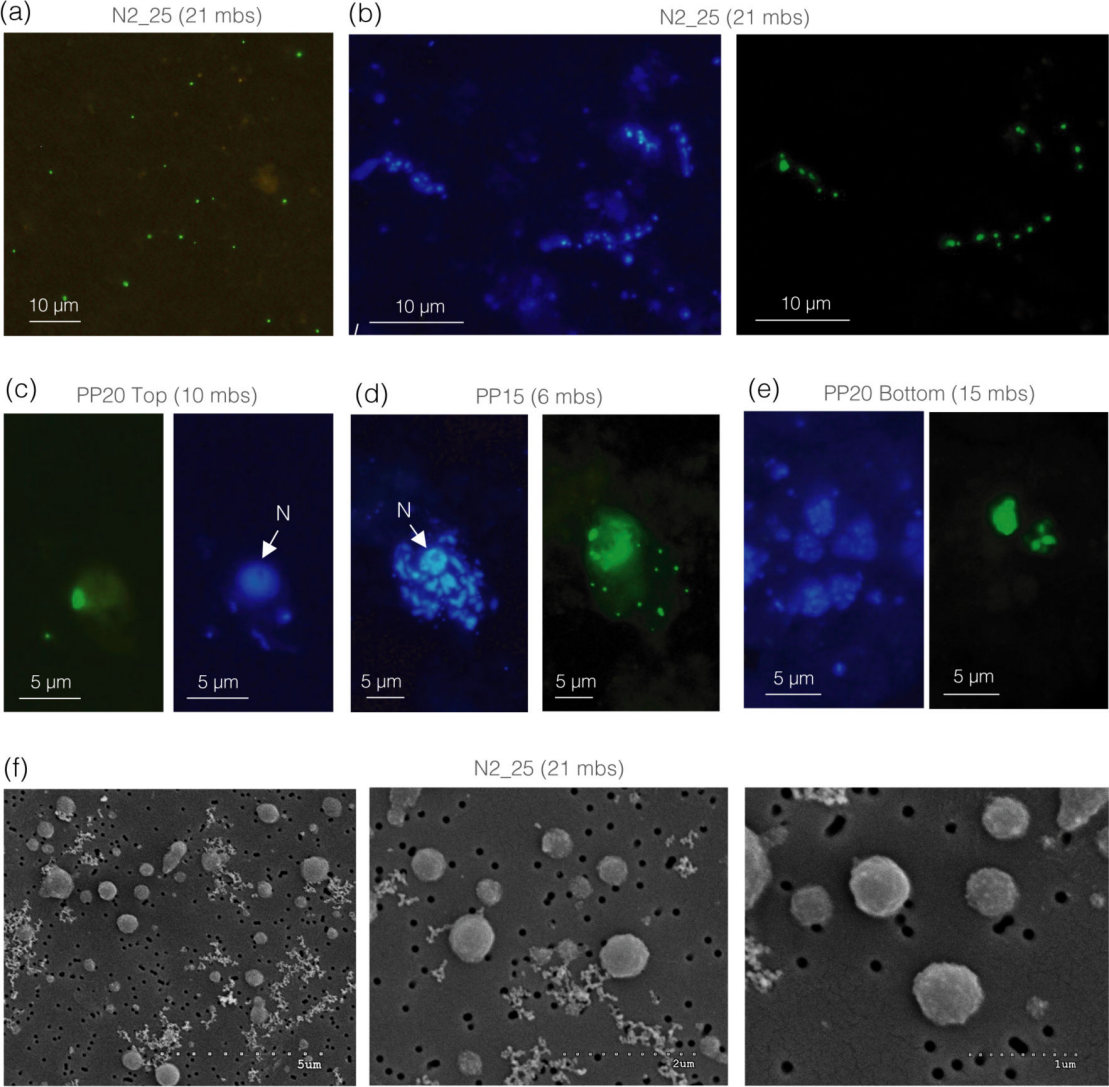
(**a–e**) Microscopic observation of cells hybridized with the OD1 CARD-FISH probe (Parcubacteria, green fluorescence). (**a**) Minute free-living cells (the prevailing cell morphology), most abundant in saline groundwater but detected across all samples. (**b–e**) Examples of OD1 cells in apparent association with other prokaryotes (**b**), eukaryotes, identified by the presence of a nucleus (**c, d**), or conforming aggregates on particles (**e**). DAPI-stained cells are shown in blue. “N” indicates eukaryotic nuclei. (**f**) Scanning electron microscopy of ultra-small cell-like particles passing through the 0.2 µm filter and retained onto the 0.1 µm filter in deep saline groundwater. mbs, meters below surface.

**Fig 4:**
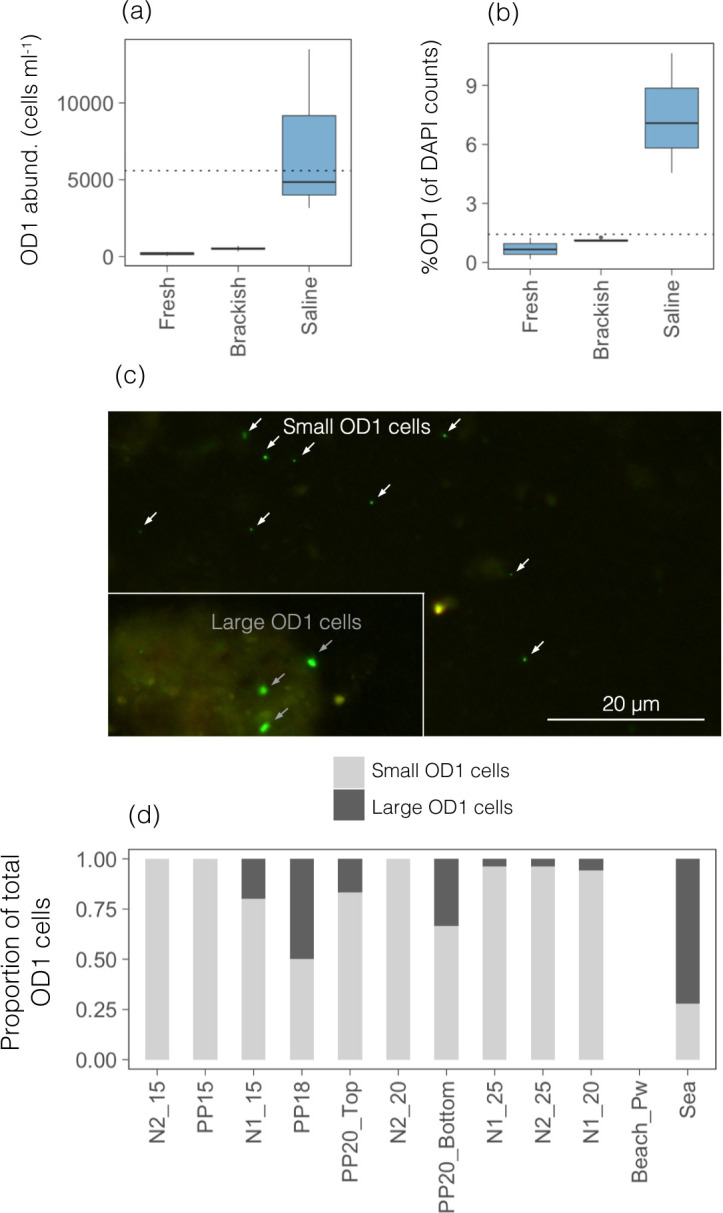
Abundance (**a**) and percentage (**b**) of Parcubacteria (OD1-labeled cells identified by CARD-FISH) across the different types of groundwater considering only the aquifer samples (*n* = 10). The dotted lines represent the OD1 cell abundance or percentage in the seawater sample, respectively, for comparison. (**c**) Micrograph showing ultra-small free-living Parcubacteria cells labeled by the CARD-FISH OD1-289 probe (white arrows), which were the dominant morphotypes among the detected hybridized cells, and apparently larger OD1 cells (gray arrows), usually found attached to particles or sediment surfaces. (**d**) Proportion of the detected OD1 cells that showed such small or large cell sizes across the studied transect. Samples are ordered by increasing salinity.

Given the dominance of Parcubacteria (OD1), we chose a CARD-FISH probe to target members of the Parcubacteria class. Minuscule labeled free-living cells were detected across all samples (Fig. 3a), but showed much higher abundances in the three deep saline sites N1_20, N1_25, and N2_25 sites (Fig. 4a and b; Fig. S4), coinciding with the clearly defined UMP populations observed in the flow cytograms of these three sites (Fig. 1a). Whereas in most fresh or brackish groundwater samplesOD1-labeled cells comprised less than 1.5% of total DAPI-stained cells (range 0.7–1.3%), in the deep marine sites the abundances and proportions were higher: 4.6% at N1_25, 7.1% at N1_20 and 10.6% in N2_25 (Fig. 4b). Image analyses of these three samples showed that OD1 cells were on average 1.7 times smaller than the small marine SAR11 cells (Fig. S5), supporting their exceptionally ultra-small sizes. In some cases, OD1-labeled bacteria were seen in apparent association with other prokaryotes, forming filaments (Fig. 3b; Fig. S6c) or with eukaryotic organisms (Fig. 3c and d; Fig. S6a and b). OD1 cells of apparently larger sizes were also detected, although we cannot discard that they are tight aggregates of small cells. These were usually attached to particles (Fig. 3e and 4c) but generally present at low abundances. These large cells comprised less than 20% (0–20%) of OD1-cells in most samples, with the exception of PP20_Bottom and PP18 where up to 33% and 50% of the OD1-positive cells had large sizes, respectively (Fig. 4d). In the surface seawater, only 1.5% of the total DAPI-stained cells were identified as OD1, the majority of which had large cell sizes.

OD1 numbers were positively related to the abundance of the UMP population estimated by flow cytometry (Spearman’s *r* = 0.72, *P* = 0.001), with total prokaryotic abundances (*r* = 0.84, *P* = 0.0001), and weakly with salinity (*r* = 0.33, *P* = 0.048). The percentage of OD1-labeled cells was positively correlated with salinity (*r* = 0.53, *P* = 0.01) and total prokaryotic abundance (*r* = 0.79, *P* = 0.0003), but negatively with nitrate concentrations (*r* = −0.48, *P* = 0.015).

SEM was used to explore in more detail the size and shape of ultra-small cells from the deep saline site with the highest concentration of the flow-cytometric UMP cells (N2_25), by concentrating cells passing through the 0.2 µm membrane onto a 0.1 µm filter (Fig. 3f). We found that the vast majority of the cells retained on the 0.1 µm filter had coccoid morphologies, but sizes were variable, with diameters ranging from 0.25 µm up to 1 µm, yet a large number of cell-like particles had diameters < 0.5 µm (Fig. 3f).

### Niche preference and drivers of coastal groundwater Patescibacteria ASVs

**Fig 5:**
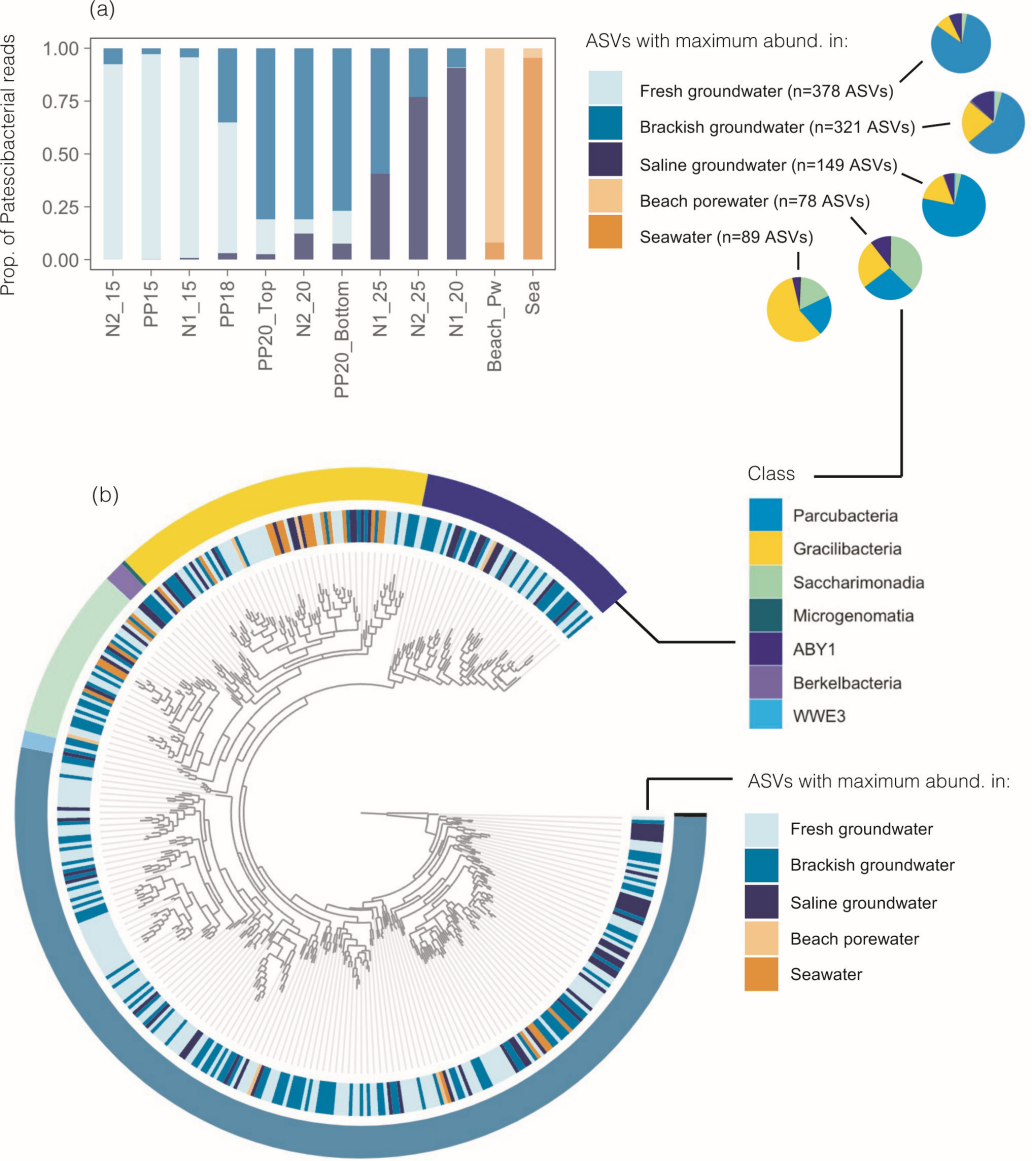
(**a**) Contribution of ASVs with different niche preferences across the studied Patescibacterial communities, categorized based on the type of habitat (fresh, brackish, saline groundwater, porewater, and seawater) where they showed their maximum relative abundance. Samples are ordered by increasing salinity. The numbers in brackets indicate the number of Patescibacteria ASVs that were identified in each case, and the pie charts indicate the composition (at the class level) of each pool of ASVs. (**b**) Phylogenetic tree of the most abundant Patescibacteria ASVs detected in this study (done with the 459 ASVs comprising >0.1% within at least one sample and 83–99% of aquifer communities). One Archaeal ASV was used as outgroup. The niche preference for every ASV and taxonomic assignation at the Class level is indicated.

We categorized ASVs depending on the salinity level (fresh, <4; brackish, 4–27; saline, >27 [48]), where they showed their maximum relative abundance in the data set, and explored their contribution to the different communities (Fig. 5a). We observed a clear replacement of ASVs with distinct habitat preferences along the salinity gradient. Fresh groundwater sites were dominated (93–97% of reads) by taxa with maximum abundances in fresh groundwater until a salinity of 3.3, after which an increased contribution (from 35% up to 81%) of taxa with brackish groundwater preference was observed. Beyond salinity 25, these were gradually replaced by ASVs with maximum abundance in saline groundwater, which increased from 41% of sequences at a salinity of 27 (N1_25) up to 91% in the most saline groundwater site (salinity 34, N1_20). Interestingly, none of these aquifer Patescibacteria were found in the porewater or surface seawater samples (Fig. 5a).

The relative contribution of Patescibacteria ASVs with preference for fresh groundwater decreased markedly with increasing salinity and decreasing nitrate, pH, and silicate, whereas bacterial groups with preference for saline groundwater showed the opposite pattern (Fig. S7a). No variable was clearly correlated to the relative abundance of groups with preference for brackish groundwater (Fig. S7a).

**Fig 6:**
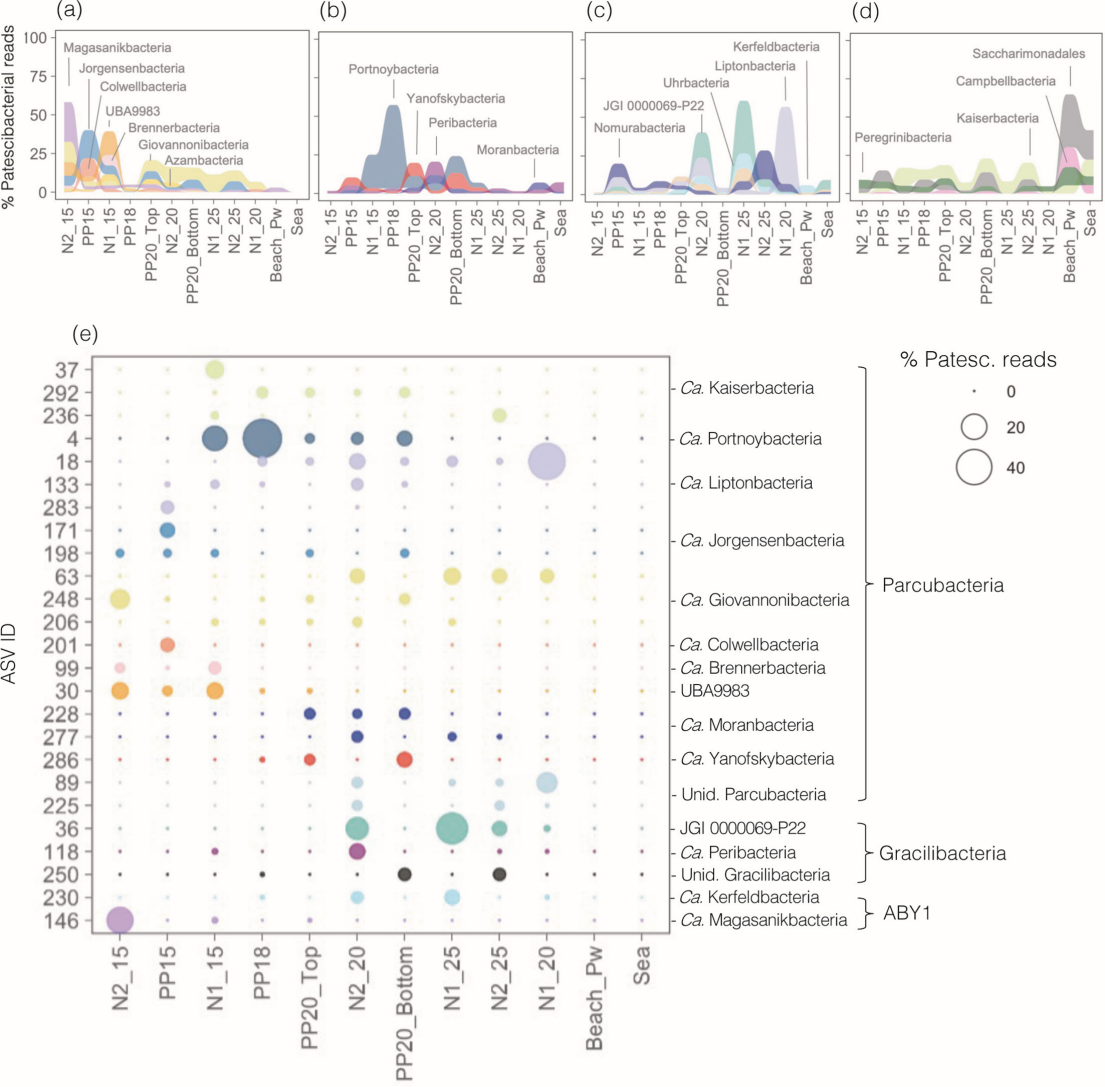
(**a–d**) Variation in the relative contribution of different groups across the studied samples depending on the portion of the transect where they were more prevalent: fresh (**a**), brackish (**b**) or saline (**c**) groundwater sites, or the beach porewater and seawater communities (**d**). (**e**) Relative abundance of the 25 most abundant Patescibacteria ASVs across the aquifer-sea continuum, color-coded by their assigned taxonomy. The size of the bubble is proportional to their relative abundance (% total Patescibacteria sequences).

We did not observe a clear taxonomic signature of the habitat preference at the class level, although higher proportions of Saccharimonadia and Gracilibacteria were found within ASVs with preference for the beach porewater or the seawater, respectively (Fig. 5a). This lack of phylogenetic coherence was also observed in a phylogeny built with the 459 most abundant ASVs, as different habitat preferences were found among closely related ASVs (Fig. 5b). In terms of specific groups, UBA9883, *Candidatus (Ca*.) Jorgensenbacteria, *Ca*. Colwellbacteria, *Ca*. Brennerbacteria, *Ca*. Azambacteria, and *Ca*. Magasanikbacteria prevailed in the fresher parts of the subterranean estuary (Fig. 6a), most showing negative correlations with salinity and positive correlations with nitrate or pH (Fig. S7b). Conversely, groups such as *Ca*. Nomurabacteria, JGI 0000069-P22, *Ca*. Uhrbacteria, and *Ca*. Liptonbacteria increased their relative abundances toward the saline groundwater sites, but this increase was restricted to individual sites enriched in nitrite rather than dependent on the salinity gradient (Fig. 6c; Fig. S7b). Groups prevailing in intermediate salinities included *Ca*. Portnoybacteria, *Ca*. Moranbacteria, and *Ca*. Yafnoskybacteria (Fig. 6b), which showed no significant correlations with salinity or other variables except for temperature in the case of *Ca*. Yafnoskybacteria. (Fig. S7b).

This niche partitioning of groups along the subterranean estuary was also observed at the ASV level: none of the 25 most abundant ASVs in the dataset showed presence throughout the entire salinity gradient (Fig. 6e), and none were detected in the beach porewater nor in the surface seawater. The most widely distributed ASV was a *Ca*. Liptonbacteria detected in seven aquifer samples (ASV_18), but the vast majority of the Patescibacterial ASVs were rare and occurred only in one sample (Fig. S8a and b). Despite this, the ASV accumulation curve tended to reach an asymptote, suggesting good sampling coverage of the Patescibacteria within the coastal aquifer (Fig. S8c).

We finally explored covariations between the Patescibacterial ASVs and other bacterial taxa, but due to the restricted distribution of most taxa, we did not detect significant relationships. At the order level, however, we observed a few significant positive correlations, such as the covariation between *Ca*. Azambacteria or *Ca*. Giovannonibacteria with Verrucomicrobiota, *Ca*. Brennerbacteria and UBA9983 with Planctomycetota, *Ca*. Jorgensenbacteria and *Ca*. Colwellbacteria with Bdellovibrionota, *Ca*. Azambacteria with Myxococcota, and *Ca*. Berkelbacteria with Alphaproteobacteria (Fig. S9).

## DISCUSSION

Despite recent advances in the study of Patescibacteria, there is little knowledge on basic features such as cell abundance, size, or environmental preferences of the different taxa. In particular, even though Patescibacteria inhabit both freshwater (12, 18, 41) and marine habitats (27, 29, 37), so far no study had explored their community assembly along salinity gradients. Here, profiting from a detailed spatial sampling of a subterranean estuary, we uncover that Patescibacteria are also abundant and diverse in coastal groundwater of varying physico-chemistry, suggesting that they may be relevant players at aquifer-sea interfaces.

### Oxygen-poor saline groundwater as a hotspot of ultra-small free-living prokaryotes

All the studied groundwater prokaryotic communities harbored a flow cytometric population of small cells (UMP) that was absent in the sampled seawater. UMP abundances were minimum in the shallowest fresh groundwater sites and increased toward brackish and saline groundwater, comprising up to 26% of total prokaryotes. At the study site, fresh groundwater flows seaward in the upper part of the aquifer, and the deeper area (<15 mbs) is affected by a seawater intrusion extending >150 m inland (49, 50). The deep saline groundwater, where UMP prevailed, is characterized by low concentrations of dissolved oxygen (0.48–0.83 mg L^−1^ at the time of sampling), the lowest pH, phosphate and nitrate concentrations, and the highest concentrations of nitrite and ammonium (48). This deep layer also harbored the highest prokaryotic abundances and heterotrophic activity throughout the aquifer when compared to fresh or brackish shallower sites (48), suggesting that these more microbially productive oxygen-poor saline groundwater sites favored the growth of ultra-small cells in the aquifer. Very few flow cytometric evidences exist of such clear UMP populations in natural waters, but Proctor et al. (41) observed that different freshwater ecosystems (including three fresh groundwater sites) harbored populations of small cells filterable through 0.4 µm that were enriched in Patescibacteria sequences. To our knowledge, however, ours is the first flow-cytometric report of UMP in coastal groundwater.

### Niche differentiation of Patescibacterial communities along the subterranean estuary

Patescibacteria represented the second most abundant phylum in the subterranean estuary based on 16S rRNA gene amplicons (48), with Parcubacteria dominating (45–87%) most aquifer Patescibacterial assemblages. Gracilibacteria, ABY1, and Saccharimonadia were also present at significant abundances. Although all these groups have previously been found in inland groundwaters (6, 12, 40), very few reports of Patescibacteria are available from coastal aquifers, where this group rarely exceeds 5% of sequences (45, 64, 65). Only recently, two studies have detected their presence in shallow coastal groundwater of different salinities, where they reach ca. 7–10% of communities (46, 47) and appear to clearly prefer porewater over sediments (46). The higher abundances of Patescibacteria (up to 22% of communities) found in our site may be due to the primer pair used, which captures higher diversity of Patescibacteria than other commonly used primers (33, 45) or to the fact that coastal aquifer microbial surveys rarely reach such deep groundwater. Future efforts using metagenomics (free of primer biases), or primers optimized for the detection of coastal aquifer Patescibacteria, will help to access a better representation of this group in subterranean estuaries, as has been recently done for other environments like, for example, wastewater treatment plants (63). However, the high degree of novelty we found among aquifer ASVs points to a very poor coverage of coastal groundwater Patescibacteria in currently available databases, which challenges primer optimization.

Salinity emerged as the most important driver of Patescibacteria, causing changes in community structure, decreases in ASV richness, and increases in UMP and OD1-cell abundances. At the study site, salinity was previously identified as a key factor determining prokaryotic community composition, abundance, cell size, and heterotrophic activity, yet Patescibacteria as a whole could not be related to salinity or any of the measured environmental parameters (48). This agrees with the observed succession of locally dominant Patescibacteria ASVs with different preferences for fresh, brackish, and saline groundwater along the subterranean estuary. Indeed, the dominance of the different groups was generally limited to one site rather than to all sites with similar salinities, supporting a high specialization of Patescibacterial taxa to the local conditions. Accordingly, most detected Patescibacterial ASVs were rare and present in just one or a few samples, and none was detected throughout the entire salinity gradient.

A similar site-specificity was also observed across distant inland aquifers in New Zealand, where very few Patescibacterial taxa were shared between sites based on 16S rRNA amplicons (12). Also, little species-level overlap was found between metagenome-assembled genomes (MAGs) across eight groundwater sites in California, supporting a differentiation of Patescibacterial communities due to local conditions (6). In our case, the most widely distributed ASV was a *Ca*. Liptonbacteria (ASV_18), present in seven aquifer brackish or saline groundwater sites. This ASV shared only 88% sequence identity to its closest representative, an unidentified Parcubacteria obtained from an aquifer sediment in Colorado (GenBank accession no. KX123508.1 [1]), confirming the lack of coastal groundwater microbial representatives in public sequence databases (45).

Although the direct effects of salinity on Patescibacteria remain unknown, their ultra-small cell size and simplified membrane structures, along with reduced transport systems and stress response pathways (9, 66), likely render them particularly susceptible to osmotic stress. Salinity is known to promote cell aggregation and biofilm formation by reducing electrostatic repulsion and stimulating the production of extracellular polymeric substances (EPS) (67). These biofilms can act as protective microenvironments, mitigating osmotic stress and facilitating metabolic cooperation among halotolerant taxa. For Patescibacteria, such strategies may be especially critical, as they might depend on syntrophic interactions to acquire essential osmoprotectants from co-occurring microbes (68). Alternatively, they may enhance their own EPS production to support aggregation and biofilm formation under saline stress. However, the rise in UMP and seemingly free-living OD1 cells in more saline groundwater suggests that some non-attached Patescibacteria groups tolerate such conditions.

Besides salinity, accumulating evidence of biogeography in Patescibacteria in inland groundwater supports community changes in response to physicochemical variations such as dissolved oxygen, pH, nitrate, and dissolved organic carbon, among others (11, 12, 24, 40). Patescibacteria have often been described from anoxic habitats (1, 69, 70), which agrees with the high UMP abundances found in our oxygen-poor brackish and saline groundwater sites. However, some taxa have been exclusively found at oxic sites or even presented positive correlations with dissolved oxygen (11, 12, 24), indicating the presence of anoxic micro-niches or that some Patescibacteria can tolerate oxic conditions. Accordingly, we found Patescibacterial sequences throughout the entire range of aquifer conditions, which span large spatial variations in salinity, nutrient and metal concentrations, and a variety of chemical reactions that determine groundwater quality (49, 71, 72). The higher abundances of the UMP population at the deep sites, though, agree with a preference of these apparently free-living ultra-small (likely Parcubacteria) cells for oxygen-poor environments, given that oxygen levels decrease with depth at the study site.

Transport from upper soils into aquifers and subsequent selection has also been suggested to explain the prevalence and success of Patescibacteria in groundwaters, as some groups such as *Ca*. Kaiserbacteraceae, Nomurabacteraceae, and UBA9983 were found to be preferentially mobilized from soils into shallow groundwater in an aquifer in Germany (24). Other studies have also shown that Patescibacteria are preferentially found in soil seepage, likely due to their ultra-small cell size (73, 74). This could explain the different Patescibacterial communities between the deep and the shallow layers of the study aquifer, as the latter are more connected to the surface (71), and perhaps also the highest Patescibacterial richness found in fresh groundwater.

### Visualization of minute free-living Parcubacteria prevailing in saline groundwater

Visualization of Parcubacteria hybridized with the OD1-289 probe showed large numbers of minuscule non-attached cells that were present across all samples but which showed much larger abundances in the three deep saline sites. Abundances of OD1-labeled cells and UMP were positively correlated (Spearman’s *r* = 0.72, *P* = 0.001), meaning that at least some of the detected UMP may be active (or at least ribosome-containing) ultra-small Parcubacteria. Parcubacteria have been suggested to be fermenters, obligately fermenting sugars to organic acids, although some seem able to degrade complex carbon substrates (7, 70, 75). Moreover, they seem to play important roles in nitrogen cycling, given that some Parcubacteria genomes encode putative components of the dissimilatory nitrate reduction to ammonia pathway (37, 76), as well as the potential for hydroxylamine oxidation (76) and nitrite reduction (40), indicating a potential role for these microorganisms in nitrification and denitrification. The study aquifer is characterized by high loads of nitrogen likely from agriculture (51), and denitrification is prevalent in the brackish aquifer portion (71, 72), so it is possible that Patescibacteria catalyze important nitrogen transformations in this aquifer. Future studies addressing the metabolic potential of coastal groundwater Patescibacteria through metagenomics or metatranscriptomics will be essential to determine their role in modulating nutrient fluxes to the sea linked to groundwater discharge.

Given that most research on Patescibacteria has largely been based on genomics, there are almost no visual proofs of these peculiar groups. Cryo-transmission electron microscopy discovered the presence of ultrasmall cells in inland aquifers, showing that they were dividing or physically interacting with other bacterial cells through pili-like structures (5, 6). In artificial environments (e.g. wastewater treatment bioreactors), some parasitic interactions between Patescibacteria and other organisms have recently been evidenced through techniques such as FISH and electron microscopy (19–21). In lakes, the application of a series of newly designed CARD-FISH probes targeting different lake Patescibacterial lineages unveiled very low abundances of individual lineages, and the size of hybridized cells fell with the range of other genome-streamlined free-living freshwater microbes (31). In our case, most OD1-labeled cells were visually much smaller than most freshwater or marine prokaryotes, and their small size was evidenced upon comparison with marine SAR11 cells, also considered ultramicrobacteria (77, 78), which were 1.7 times larger than the observed Parcubacteria. We thus believe that this is the first visual evidence of ultra-small sized free-living Parcubacteria, and their abundances might be underestimates because the OD1 probe targets only a fraction of Parcubacteria (Fig. S1) and because cells were collected onto 0.2 µm and some might have passed through. Generally, however, 0.2 µm filters were quickly clogged and most small prokaryotes were likely retained on this fraction. SEM of 0.2 µm filtered groundwater collected onto 0.1 µm further showed the presence of coccoid cell-like particles spanning diameters ranging from 0.25 µm up to 1 µm, but with many being smaller than 0.5 µm diameter. These sizes fall within the size range previously estimated for Patescibacteria based on cryo-transmission electron microscopy (0.009 ± 0.002 µm^3^ [5] and <0.5 µm diameter [6]) or fluorescence-activated cell-sorting (<0.25 µm diameter [8]), and approximate the lower theoretical limits for cellular life (78, 79).

The fact that most hybridized Parcubacteria were small and un-attached across most aquifer samples agrees with the reported enrichment of Parcubacteria in the <0.2 µm size fraction (6, 11, 24) and with findings suggesting that many subsurface Patescibacteria may not form specific physical associations with other microorganisms (8, 11). However, we also found evidence of particle-attached or symbiotic lifestyles. For example, OD1 cells of larger apparent sizes were also detected, usually attached to non-living particles, but in a few occasions, ultra-small OD1 cells were seen in apparent association with other prokaryotes or eukaryotes. Parcubacteria were initially proposed to be symbionts based on their small genomes and genomic signatures such as the lack of biosynthetic capacities, as well as the presence of attachment and adhesion proteins (13), and microscopy has provided evidence for a few host-associated Parcubacteria, the *Ca. Sonnebornia yantaiensis*, endosymbiont of the freshwater *Paramecium bursaria* (14) and *Ca*. Nealsonbacteria, potential ectosymbionts of methanogenic archaea (*Methanothrix*) discovered in benzene-degrading enrichment cultures (80). Notably, although the FISH images in the latter study of *Methanothrix* symbionts, as well as those reported in a wastewater treating bioreactor (21), closely resemble the observed associations between OD1 cells and prokaryotic filaments in our aquifer (Fig. 3b; Fig. S6c), we did not detect sequences of these archaeal hosts. Interestingly, a parasite of the archaeon *Methanospirillum hungatei,* which was until recently classified within Parcubacteria, has now been cultivated and described as a new species (*Minisyncoccus archaeiphilus*) within the newly proposed class Minisyncoccia and phylum Minisyncoccota (20). This highlights the dynamic and still-evolving taxonomy of Patescibacteria, as well as the growing evidence for host-associated lifestyles within this group.

The detection of different lifestyles within Parcubacteria in our site agrees with the CARD-FISH results of Chiriac et al. (31) in lakes, who observed free-living and particle-associated cells even within a given genus. Similarly, Gios et al. (12) found that most 16S rRNA gene sequences of ultra-small groups (including Patescibacteria) were present both in the planktonic and particle-attached compartments across different aquifers. Chaudhari et al. (11) concluded that, although most Patescibacteria detected across six groundwater wells had the capacity to attach to other cells, this attachment may not be very specific or during long time periods, but rather long enough to exchange supplies. It is finally possible that OD1 cells may have detached from their hosts during sample processing, as shown for other bacterial symbionts (81), but the clear flow cytometric UMP population seems to suggest that at least a fraction of the Patescibacteria displayed a free-living lifestyle, particularly in deep saline groundwater sites.

In summary, our results present new insights on Patescibacterial abundance, diversity, niche preferences, and distribution in an intricated coastal groundwater system with strong environmental gradients, which hosts diverse Patescibacterial assemblages with a high degree of novelty beyond the species level. We show a replacement of Patescibacteria taxa with varying preferences for fresh, brackish, and saline groundwater, reflecting their versatility to occupy ample groundwater environmental gradients. Whereas this likely results in Patescibacteria communities having different roles in groundwater transformations throughout the aquifer, their ecological roles and relevance in shaping groundwater quality and nutrient fluxes to the sea remain to be addressed. Using CARD-FISH, we were able to visualize ultra-small free-living Parcubacteria, which were abundant in oxygen-poor deep saline groundwater, and also potential host- or particle-associated Parcubacteria. Our findings suggest an overlooked importance of Patescibacteria in coastal aquifers and highlight these as ideal ecosystems to gain insight into the ecology of this enigmatic group.

## Data Availability

DNA sequences and associated metadata had been previously deposited in the European Nucleotide Archive (ENA) under accession number PRJEB52186 (48). The environmental metadata can be found as Table S4 in reference 48 and the non-rarefied Patescibacteria ASV and taxonomic tables are provided as Tables S1 and S2.

## SUPPLEMENTAL MATERIAL

Supplemental figures msystems.01125-25-s0001.pdf Fig. S1-S9.

Tables S1 and S2 msystems.01125-25-s0002.xlsx ASV and taxonomy tables.

ASM does not own the copyrights to Supplemental Material that may be linked to, or accessed through, an article. The authors have granted ASM a non-exclusive, world-wide license to publish the Supplemental Material files. Please contact the corresponding author directly for reuse.
