# Supplementary material for: Diverse Patescibacteria assemblages and prevalence of ultra-small free-living Parcubacteria along a subterranean estuary
Source: mSystems. 2025 Oct 20;10(11):e01125-25. doi: 10.1128/msystems.01125-25 (PMC12625730; doi:10.1128/msystems.01125-25)

SUPPLEMENTARY FIGURES FOR:

Diverse Patescibacteria assemblages and prevalence of ultra-small free-living Parcubacteria along a subterranean estuary

Clara Ruiz-González, Catalina Mena, Francisco M. Cornejo-Castillo, Daniel Romano-Gude, Néstor Arandia-Gorostidi, Josep M. Gasol

Institut de Ciències del Mar (ICM-CSIC), E08003 Barcelona, Spain

Table of contents

Figure S1. Coverage of CARD-FISH probe

Figure S2. Novelty within detected Patescibacteria ASVs

Figure S3. Patescibacteria ASVs richness in groundwater samples

Figure S4. Examples of images showing free-living Parcubacteria

Figure S5. Comparison of cell areas between Parcubacteria and SAR11

Figure S6. Examples of images showing Parcubacteria attached to other organisms

Figure S7. Correlations between groups and environmental conditions

Figure S8. Occurrence, rarity and ASV accumulation curve.

Figure S9. Correlations between Patescibacteria and other prokaryotes

Figure S1 | Diversity coverage of the OD1-289 CARD-FISH probe in Parcubacteria and the different Parcubacterial groups. The diversity coverage was tested *in silico* using the online tool TestProbe (Klindworth *et al*. 2013), by comparing the sequences captured by the probe against the SILVA 138.2 taxonomic database, allowing for zero mismatches (i.e. no differences between the compared sequences). Except for a single matched sequence of Gracilibacteria (*, ACCN: EU101254.1, not detected in our dataset), no unspecific binding among other groups was detected, suggesting that all OD1 detected cells were Parcubacteria.

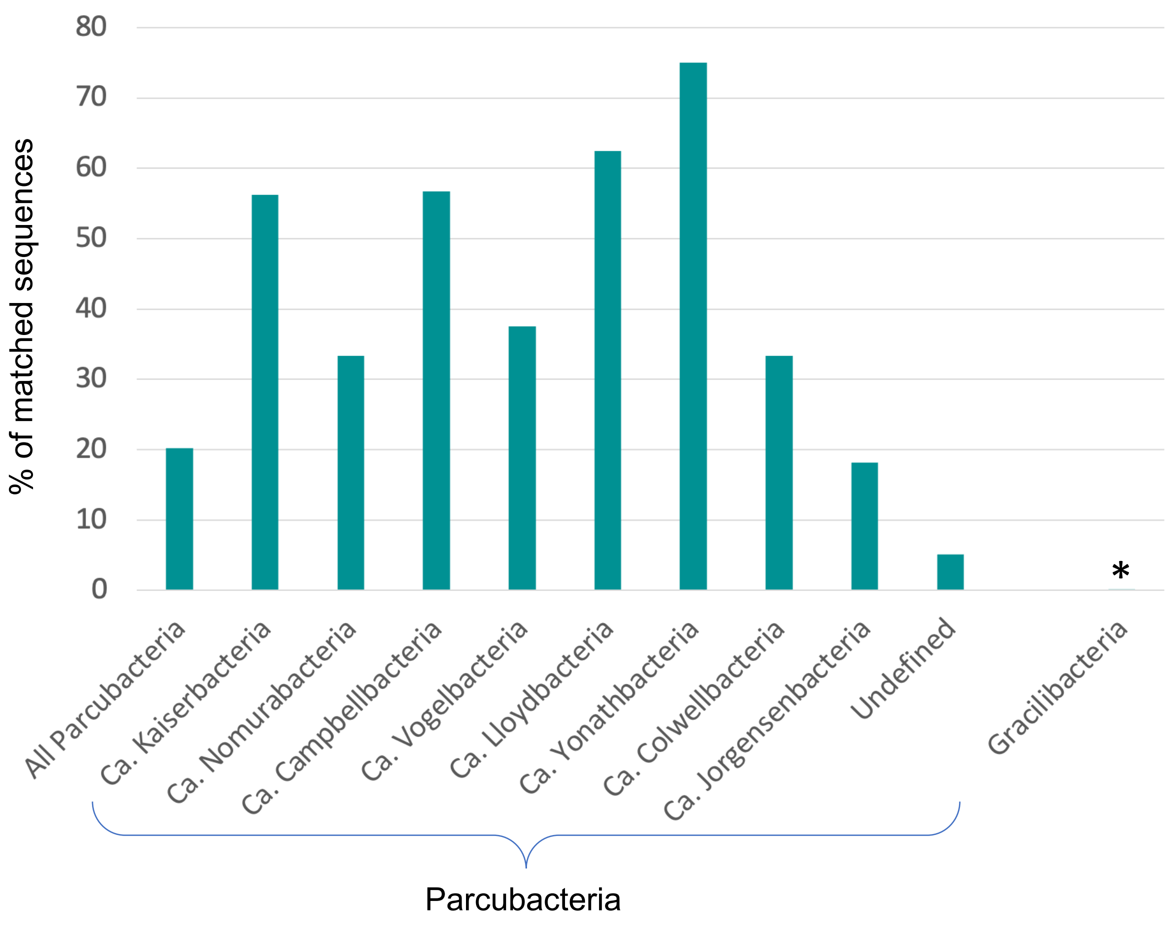

Figure S2 | Percent similarity of the 1015 Patescibacterial ASVs and their closest match in the Silva database (v. 138.2). The vertical lines indicate percentages of sequence identity that have been proposed as approximate thresholds for distinguishing genera (≤ 94.5%), families (≤ 86.5 %), orders (≤82.0 %), classes (≤78.5 %), and phyla (≤75%) following Yarza et al. (2014).

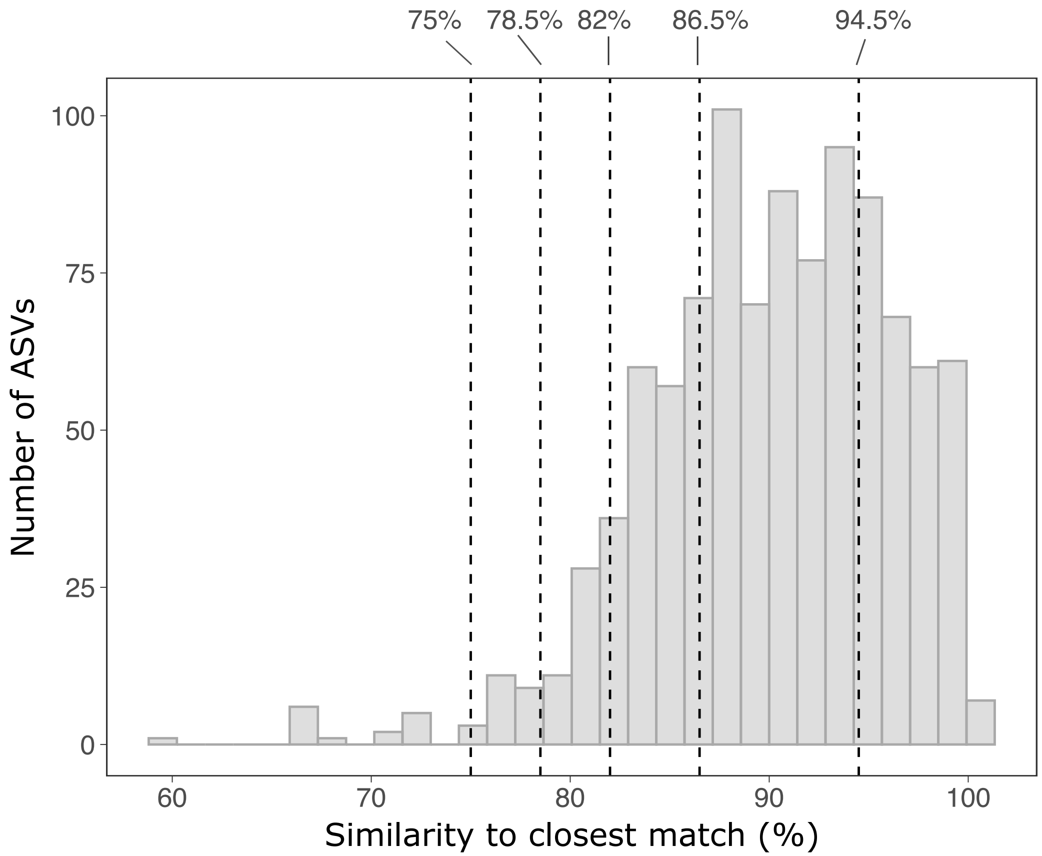

Figure S3 | Variations in taxonomic richness within the studied Patescibacteria communities. The dashed and dotted lines represent the UMP abundance or percentage in the beach porewater and seawater samples, respectively, for comparison.

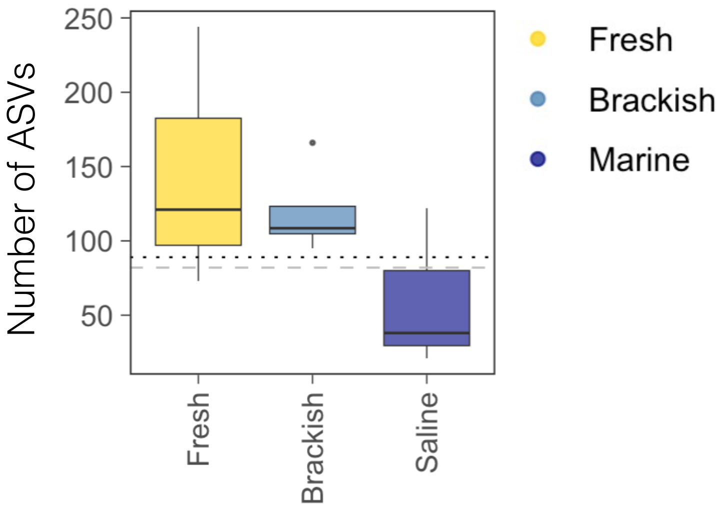

Figure S4 | Examples of microscopic images of minute cells hybridized with the OD1-289 CARD-FISH probe (Parcubacteria, green fluorescence) showing an apparent free-living lifestyle. DAPI-stained cells are shown in blue.

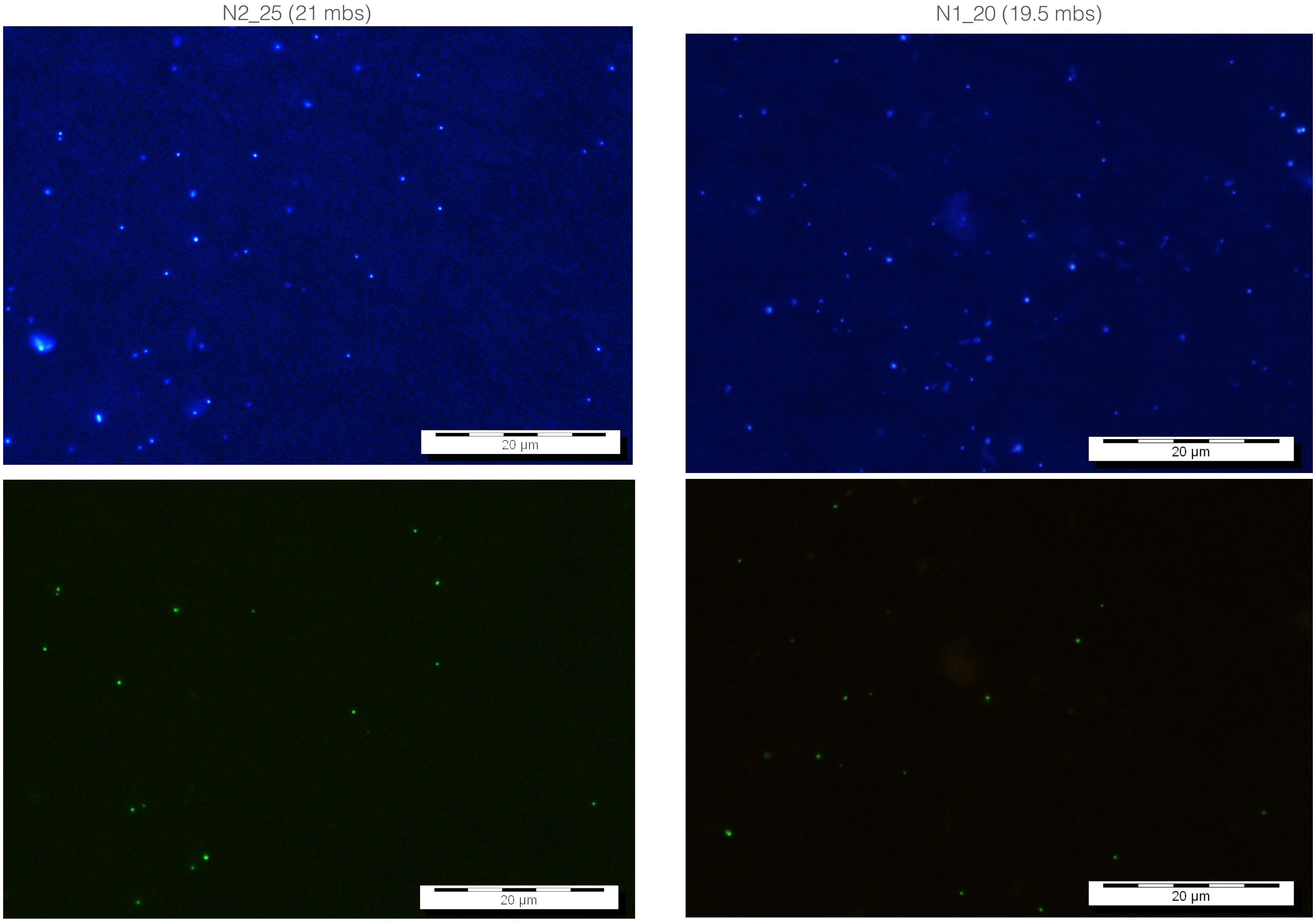

Figure S5 | Estimated size of cells hybridized with the OD1-289 CARD-FISH probe (Parcubacteria). (a-c) Cell sizes (as cell area in pixels, px) of the three deepest saline groundwater samples. Note that in the three cases most cells were small. Vertical dashed lines indicate the geometric mean (44.14, 48.01 and 48.58 px for samples N2_25, n1_20 and n1_25, respectively). (d-e) Comparison between cell size of OD1-hybridized cells from this study (cells measurements of the three samples analyzed) and that of small-sized SAR11 cells from an oligotrophic coastal site (Blanes Bay Microbial Observatory, Spain). Vertical dashed lines in (e) indicate the geometric mean (40.85 px for OD1 and 67.66 px for SAR11), supporting that OD1 cells are smaller than SAR11.

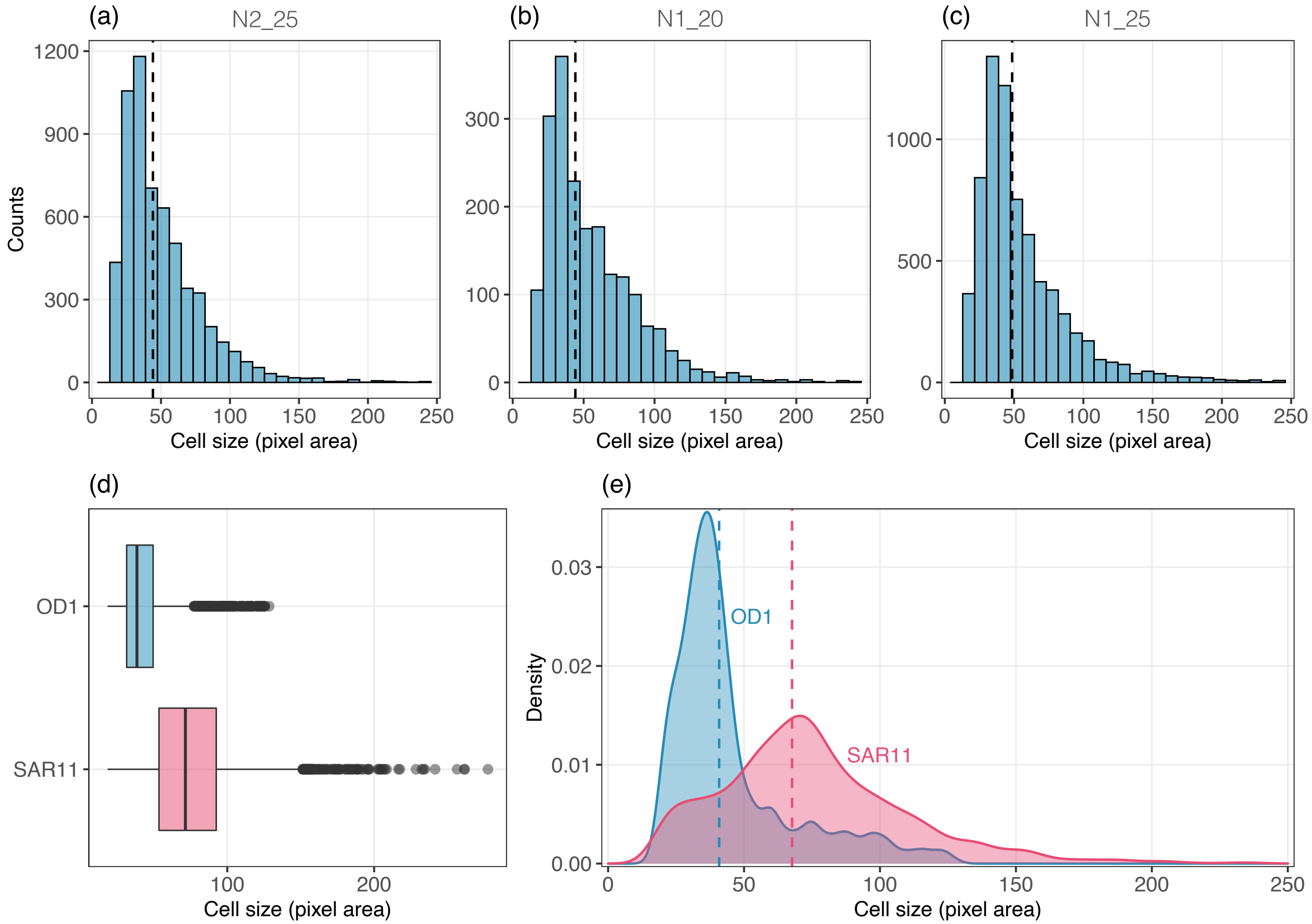

Figure S6 | Examples of microscopic images of cells hybridized with the OD1-289 CARD-FISH probe (Parcubacteria, green fluorescence) in apparent association with eukaryotes (a,b) or other prokaryotes (c). DAPI-stained cells are shown in blue. "N" indicates eukaryotic nuclei.

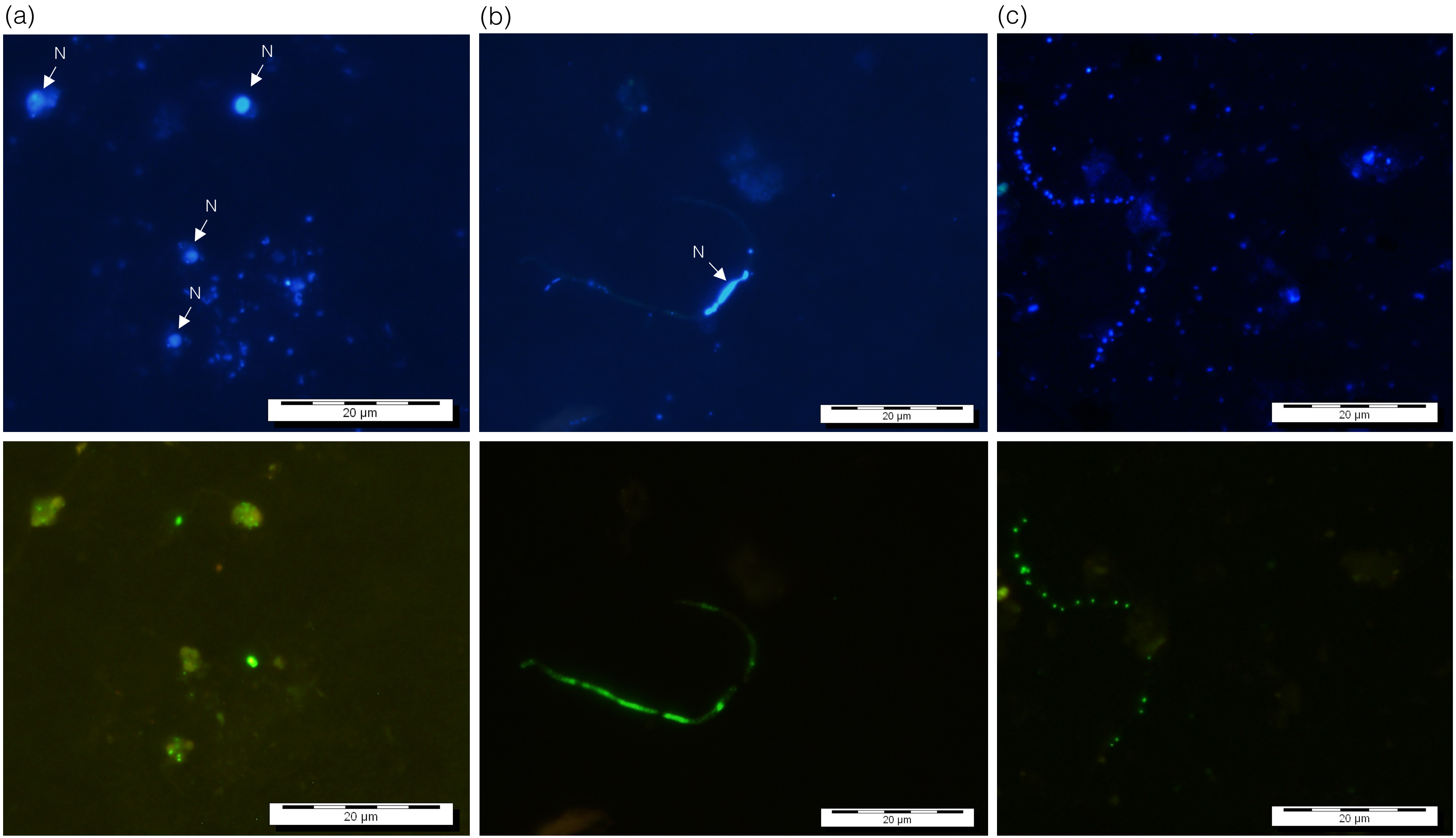

Figure S7 | Hierarchically clustered heatmap of correlations between the abundance of the three pools of groundwater ASVs identified based on their maximum abundances (a, see Figure 5) or the different taxonomic orders shown in Figure 6 (b) and the measured physico-chemical conditions, considering only the aquifer samples (n=10). The color gradient indicates the Spearman's rank correlation coefficients (rho values). The asterisks (*) indicate significant relationships (*p<0.05, **p<0.01 and ***p<0.001). DO, dissolved oxygen (mg L^{-1}); Sal, groundwater salinity; Temp., groundwater temperature (°C); nutrient concentrations in μM .

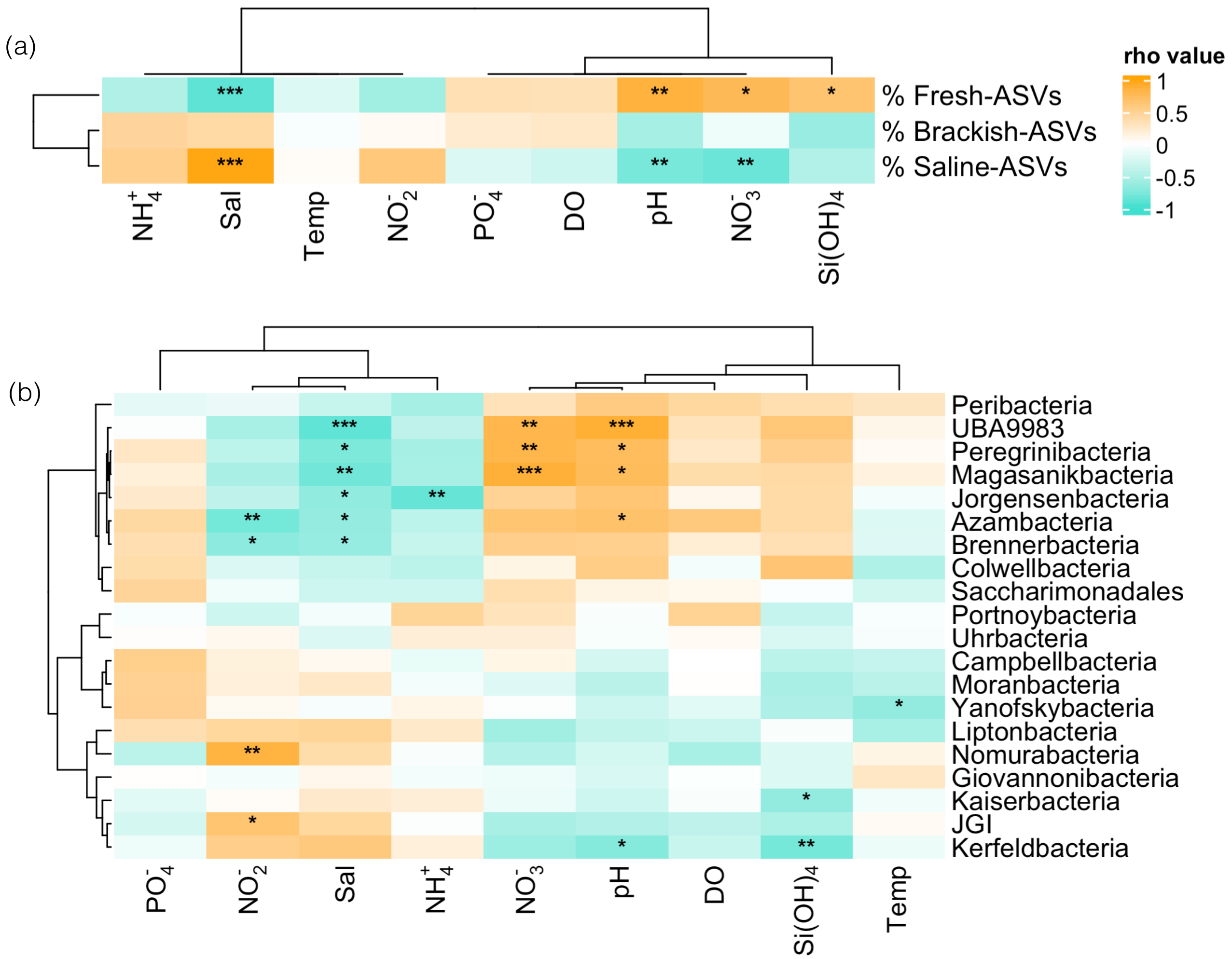

Figure S8 | Occurrence (i.e., number of samples in which a given ASV is present, (a)) and mean abundance (b) of all the ASVs identified within Patescibacteria. (c) ASV accumulation curve considering only groundwater samples (n=10).

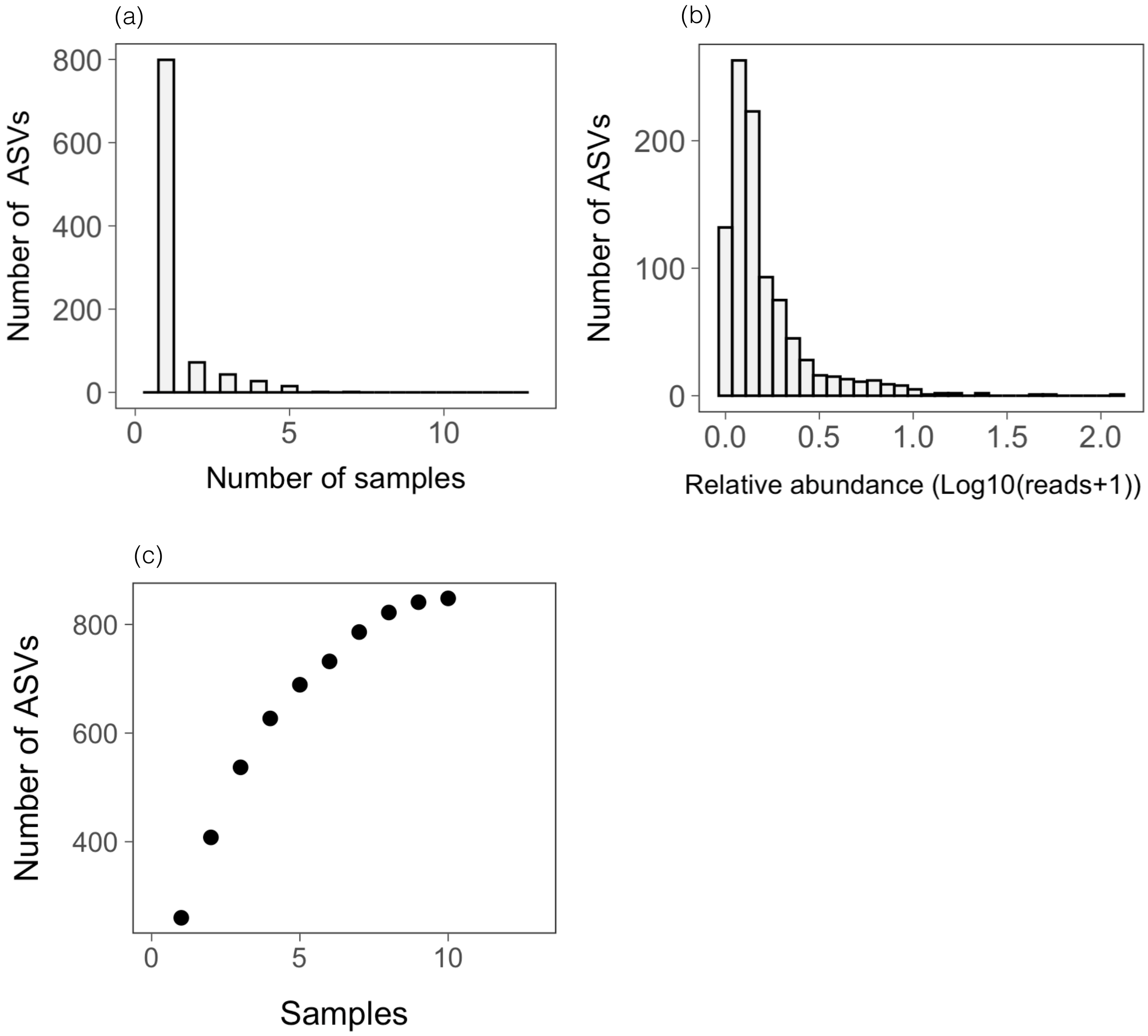

Figure S9 | Significant correlations between the relative abundances (% of total reads) of different Patescibacterial groups and bacterial phyla or class considering only the aquifer samples (n=10), color-coded by salinity level. The blue lines are linear regressions. Shaded areas represent the 95% confidence intervals.

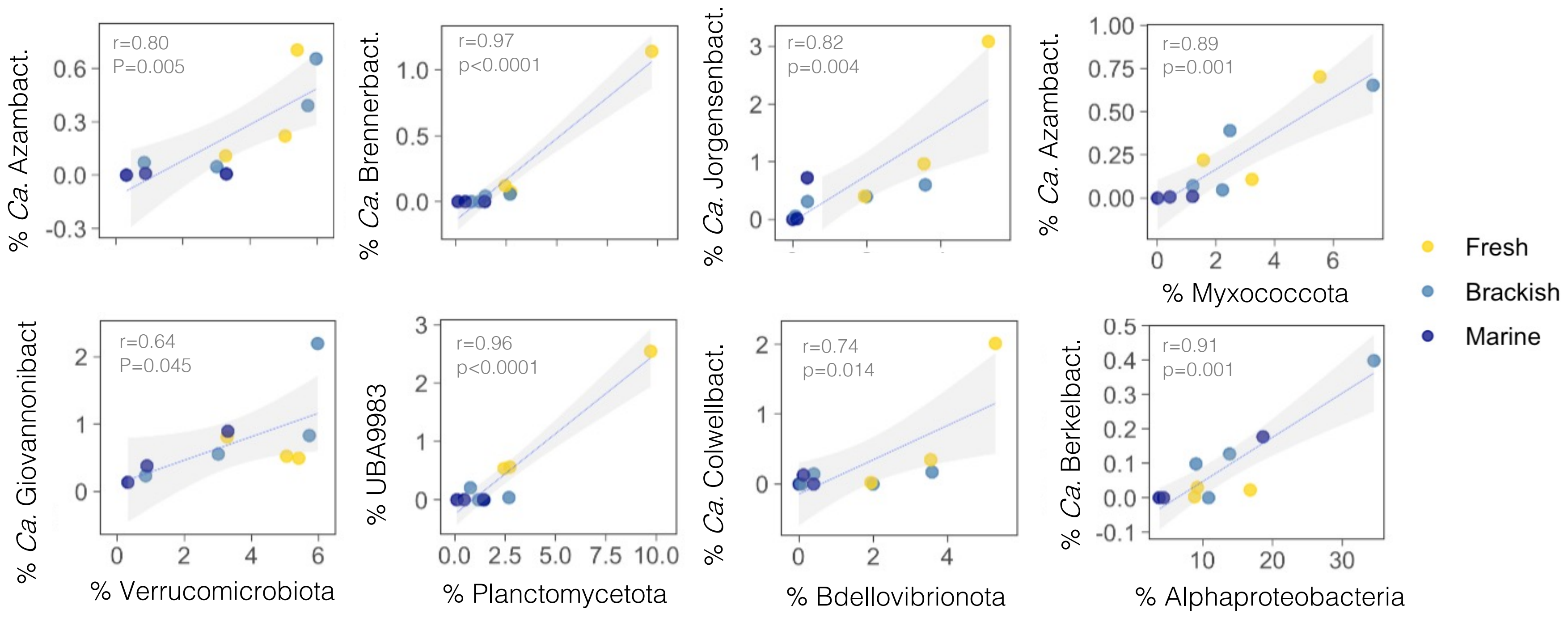

Supplement: Supplemental figures — Fig. S1-S9. [file msystems.01125-25-s0001.pdf]
